# In Vitro Tumor Cell Growth Inhibition Induced by *Lophocereus marginatus* (DC.) S. Arias and Terrazas Endophytic Fungi Extracts

**DOI:** 10.3390/ijerph18189917

**Published:** 2021-09-21

**Authors:** Jesica M. Ramírez-Villalobos, César I. Romo-Sáenz, Karla S. Morán-Santibañez, Patricia Tamez-Guerra, Ramiro Quintanilla-Licea, Alonso A. Orozco-Flores, Ricardo Romero-Arguelles, Reyes Tamez-Guerra, Cristina Rodríguez-Padilla, Ricardo Gomez-Flores

**Affiliations:** 1 Departamento de Microbiología e Inmunología, Facultad de Ciencias Biológicas, Universidad Autónoma de Nuevo León, San Nicolás de los Garza 66455, Mexico; jesicamrv_901@hotmail.com (J.M.R.-V.); karlita_moran@hotmail.com (K.S.M.-S.); patamez@hotmail.com (P.T.-G.); lacxelo@gmail.com (A.A.O.-F.); ricardoromeroarguelles@gmail.com (R.R.-A.); reyes.tamezgr@uanl.edu.mx (R.T.-G.); crrodrig07@gmail.com (C.R.-P.); 2 Departamento de Química, Universidad Autónoma de Nuevo León, San Nicolás de los Garza 66455, Mexico; rquintanilla.uanl@gmail.com

**Keywords:** antitumor, endophytic fungi, Cactaceae, *Lophocereus marginatus*, natural sources, environmentally friendly, Mexican plants, medicinal plants

## Abstract

Endophytic fungi have become potential sources of antitumor agents, particularly against antineoplastic-resistant cancer cells, with marginal or nil adverse effects for the oncological patient. Endophytic fungi were isolated from stems of the *Lophocereus marginatus* cactus, commonly found in Mexico. Methanol extracts were then obtained from fungus liquid cultures and their effects on tumor cell growth against murine lymphoma (L5178Y-R), human colorectal adenocarcinoma (HT-29), and human breast cancer (MCF-7) cells were evaluated at concentrations ranging from 31 µg/mL to 250 µg/mL via the colorimetric 3- [4,5-dimethylthiazol-2-yl]-2,5-diphenyltetrazoliumbromide reduction assay, using monkey kidney epithelial (MA-104) and human peripheral mononuclear (PBMC) cells as controls. Furthermore, we obtained the IC_50_ and the selectivity index (SI) was calculated from the IC_50_ ratio of normal and tumor cells. In addition, molecular identification of fungi showing cytotoxic activity was determined, using internal transcribed spacer molecular markers. PME-H001, PME-H002, PME-H005, PME-H007, and PME-H008 filamentous fungus strain extracts showed significant (*p* < 0.05) tumor cell growth inhibition. In particular, they significantly (*p* < 0.05) inhibited L5178Y-R cell growth, whereas the least susceptible cell line was HT-29. The endophytic strain PME-H008 of *Cladosporium* sp. caused the highest growth inhibition percentage against L5178Y-R and HT-29 cells with 96.6% (*p* < 0.01) and 42.5% (*p* < 0.05) respectively, and the highest SIs against L5178Y-R cells with 2.4 and 2.9 for MA-104 and PBMCs, respectively, whereas the PME-H005 extract showed SIs of 2.77 and 1.5 against MCF-7 and L5178Y-R cells, respectively, as compared with PBMCs. In addition, the endophytic strain PME-H007 of *Metarhizium anisopliae* caused the highest percentage of growth inhibition (*p* < 0.01) against MCF-7 cells with 55.8% at 250 µg/mL. We demonstrated in vitro antitumor effects of *L. marginatus* endophytic fungi. Further research will involve the isolation and in vivo testing of bioactive compounds.

## 1. Introduction

In 2020, around 9.9 million deaths by cancer were reported in the GLOBOCAN database [1], despite the existence of a variety of therapeutic approaches [2]. In particular, chemotherapy represents a common treatment, but severe side effects to the patients are usually observed. In addition, the emergence of antineoplastic-resistant cancer cells has become one of the main causes of treatment failure [3]. Therefore, it is essential to search for new drugs with marginal or no adverse effects for the oncological patient [4], and to which resistance has not been developed.

Endophytic fungi have gained relevance in biotechnology as potential sources of new compounds with antitumor activity. Their rapid growth, culture conditions, high cell density, easy genetic manipulation, and the possibility of scaling the production of compounds at an industrial level make them candidates for obtaining more efficacious anticancer drugs [5].

It has been proposed that the isolation of endophytic fungi, involving the selection of plants with ethnobotanical use, as well as those that have developed strategies for survival or growth under extreme environments [6], may lead to the discovery of endophytes that produce novel bioactive compounds. In this regard, arid-zone plants such as cacti establish symbiotic relationships with different microorganisms, from which enzymes [7], antimicrobials [8], and anticancer compounds such as bikaverine [9] and triterpenes of the 24-homo-30-nor-cycloartane class [10] have been isolated. However, the biotechnological potential of fungi isolated from dessert plants has not been fully investigated [11].

*Lophocereus marginatus,* also known as *Pachycereus marginatus*, is a species of cactus endemic to Mexico and belonging to the Cactaceae family. It is popularly known as chilayo, organ cactus, or malinche [12]. In traditional medicine, it has been used for the treatment of gastrointestinal diseases [13] and diabetes [14]. Recent studies have demonstrated the antimicrobial [15] and anticancer activity of *L. marginatus* extracts in in vitro [16,17] and in vivo models [18]. However, the anticancer potential of *L. marginatus* endophytic fungi have not yet been reported. The aim of the present study was to isolate and evaluate the antitumor potential of *L. mariginatus* endophytic fungi against murine and human cancer cell lines.

## 2. Materials and Methods

### 2.1. Plant Material

*L. marginatus* stems were collected in General Escobedo, Nuevo León, México (25°47′15.7″ N 100°17′32.6″ W) in February 2020. This cactus was identified by M.Sci. María del Consuelo González de la Rosa, Chief of the Herbarium of Facultad de Ciencias Biológicas at Universidad Autónoma de Nuevo León, México, with voucher specimen number 025588.

### 2.2. Isolation and Morphological Characterization of L. marginatus Endophytic Fungi

Stems were rinsed with tap water to eliminate dust and other contaminating material, and subjected to a disinfection protocol to remove epiphytes, which consisted of washing with 70% ethanol for 1 min, 2.5% sodium hypochlorite for 3 min, 70% ethanol for 30 s, and two rinses with sterile distilled water and one with PBS [19]. For the isolation of endophytic fungi, the previously disinfected plant tissue was cut into small pieces (~0.5 cm^2^). One part was placed on the surface of Petri dishes containing potato dextrose agar (PDA; Difco, Detroit, MI, USA), Sabouraud dextrose agar (SA; Difco), and water agar containing 60 mg/L penicillin and 100 mg/L streptomycin (Life Technologies, Grand Island, NY, USA) to inhibit microbial growth, and the other part was ground in PBS in a sterile mortar. Next, 100 µL of the sample was inoculated into the aforementioned culture medium by plate dispersion and the last wash with PBS was used as a negative growth control. Plates were then incubated at 20 °C for four weeks. Morphological characterization was determined from monosporic cultures of the isolates in PDA, with radial growth, shape, size, color, edge, and type of mycelium recorded. Isolated fungi were registered with isolate codes PME-H00#.

### 2.3. Fermentation and Production of Methanol Extracts

For the extraction of secondary metabolites, 1 cm^2^ fresh culture fragments were individually inoculated into 250 mL flasks with 125 mL of potato and dextrose broth (PDB; Difco) and incubated for 30 d at 20 °C and 150 rpm (ET-4200, Tecnal Incubator, São Paulo, Brazil). After incubation, mycelium was separated by filtration and dried at 60 °C, after which it was subjected to an extraction via maceration with methanol. Solvent was then removed with a rotary evaporator (Buchi R-3000; Brinkman Instruments, Inc., Westbury, NY, USA). Extracts were dissolved in dimethyl sulfoxide (DMSO; Sigma-Aldrich, St. Louis, MO, USA) at a final concentration of 25 mg/mL and kept at 4 °C until use.

### 2.4. Cell Lines and Culture Conditions

The cell lines used in this study were the murine lymphoma line L5178Y-R (ATCC CRL-1722), the human colorectal adenocarcinoma line HT-29 (ATCC HTB-38), the human breast cancer line MCF-7 (ATCC HTB-2), and the monkey kidney epithelial cell line MA-104 (ATCC CRL-2378.1). Peripheral blood mononuclear cells (PBMCs) were obtained from 20 mL to 30 mL (three experiments were performed) samples of blood from a healthy volunteer donor, using Ficoll-Paque PLUS (GE Healthcare Life Sciences, Pittsburgh, PA, USA). Cells were maintained in RPMI-1640 medium (Life Technologies) supplemented with 10% fetal bovine serum (FBS; Life Technologies) and 1% antibiotic–antifungal solution (Life Technologies), whereas MCF-7 cells were grown in Dulbecco’s modified Eagle medium (DMEM; Life Technologies) supplemented with 10% FBS and 1% antibiotic–antifungal solution (Life Technologies) (complete culture medium). Cells were cultured at 37 °C in an atmosphere of 5% CO_2_.

### 2.5. Effect of Endophytic Fungus Strain Extracts on Murine and Human Tumor Cell Growth

L5178Y-R, HT-29, MCF-7, and MA-104 cell suspensions were cultured at a density of 1 × 10^4^ cells/well and PBMCs at 1 × 10^5^ cells/well into flat-bottomed 96-well plates (Corning Incorporated, Corning, NY, USA) in complete culture medium. After 24 h of incubation, cells were treated with 31 µg/mL, 62.5 µg/mL, 125 µg/mL, and 250 µg/mL of endophytic fungus methanol extracts for 48 h at 37 °C in 5% CO_2_. Tumor cell growth was then evaluated using the colorimetric 3- [4,5-dimethylthiazol-2-yl]-2,5-diphenyltetrazoliumbromide (MTT; Affymetrix, Cleveland, OH, USA) reduction assay by adding 15 µL of MTT (0.5 mg/mL final concentration) and incubating at 37 °C for an additional 4 h. Formazan crystals were dissolved with DMSO, and optical densities (OD) were measured at 570 nm in a MULTISKAN GO microplate reader (Thermo Fisher Scientific, Waltham, MA, USA). Cell growth inhibition percentage was calculated as follows: % Growth inhibition = 100 − [(OD_570_ in extract-treated cells/OD_570_ in untreated cells) (100)], using 0.05 µg/mL vincristine sulphate (VC; Hospira, Warwickshire, UK) as a positive control. Logarithmic scale concentrations were plotted against % cytotoxicity to determine IC_50_ values, which were used to determine the selectivity index (SI). This index was calculated by dividing the IC_50_ of normal cells by that of tumor cells [20].

### 2.6. Molecular Identification of L. marginatus Endophytic fungi

Genomic DNA extraction was performed from monosporic cultures, using cetyl trimethylammonium bromide (CTAB; Sigma-Aldrich, St. Louis, MO, USA) as reported by Kuramane-Izioka [21]. Purified DNA was then subjected to a PCR with the universal markers ITS1 (5′-TCCGTAGGTGAACCTGCGG-3′) and ITS4 (5′-TCCTCCGCTTATTGATATGC-3′) in a volume of 50 µL, using the Ruby Taq Master mix 2X (Jena Bioscience, Jena, Germany), 100 ng of the DNA template, and 0.25 µM of each primer. The amplification program consisted of a denaturation cycle of 95 °C for 5 min, 35 cycles of 94 °C for 30 s, 60 °C for 45 s, and 72 °C for 90 s, followed by a final extension of 72 °C for 8 min [22]. The PCR product was purified with the Agarose Gel Extraction kit (Jena Bioscience). The product was then sequenced with the ABI PRISM 310 TM Genetic Analyzer sequencer at the Synthesis and Sequencing Unit of the Institute of Biotechnology (IBT) of the UNAM in Cuernavaca Morelos. Sequence analysis was performed using the National Center for Biotechnology Information database via the BLASTn tool [https://blast.ncbi.nlm.nih.gov/Blast.cgi (accessed on 9 February 2021)] to identify the closest fungal species. The assigned names of fungal isolates were based on the BLAST homology percentages.

### 2.7. Statistical Analysis

Cytotoxicity results were expressed as mean ± SEM of triplicate determinations from three independent experiments. Level of significance was evaluated by the Dunnet’s *t* test. IC_50_ values were reported with 95% confidence intervals (95% CI). Statistical analyses were performed using the Graph Pad Prism 7 program.

## 3. Results

### 3.1. Isolation of L. marginatus Endophytic Fungi

We isolated filamentous fungi from *L. marginatus* stems, but only data of endophytic fungus strains with cytotoxic activity are shown (strains PME-H001, PME-H002, PME-H005, PME-H007, and PME-H008). Isolates were morphologically characterized, with most of them showing circular shape, filamentous edge, and flat mycelium. Radial growth at 3 d and 7 d was from 5.1 mm to 8.5 mm and 9.9 mm to 22.6 mm, respectively. Isolates first fermented in PDB, after which biomass methanol extraction was performed, obtaining yields ranging from 5.2% to 12% (Table 1).

### 3.2. Effect of L. marginatus Endophytic Fungi Extracts on Tumor Cell Growth

**Figure 1:**
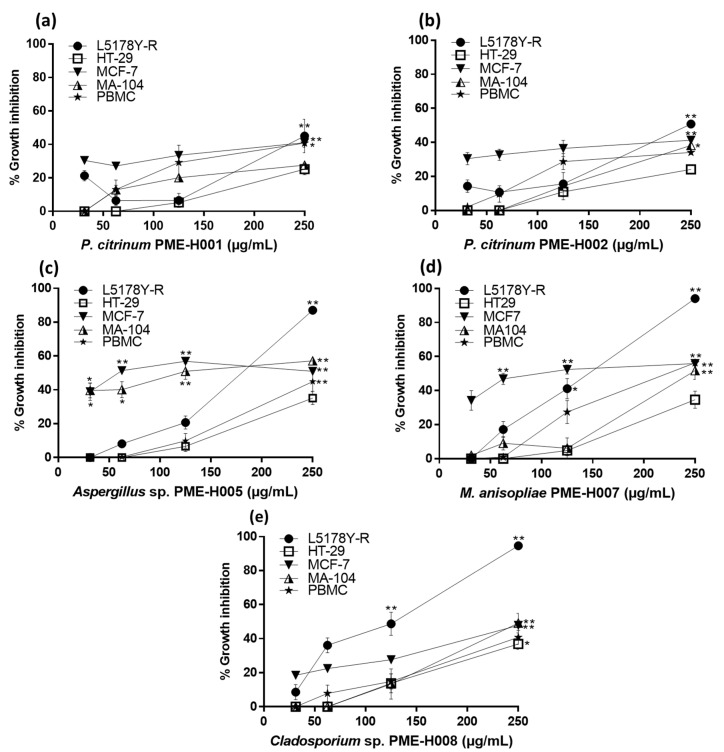
Tumor growth inhibition by *L. marginatus* endophytic fungus methanol extracts. L5178Y-R, HT-29, MCF-7, and MA-104 cells at 1 × 10^4^ cells/well and PBMCs at 1 × 10^5^ cells/well were incubated for 24 h and treated for 48 with 31 µg/mL to 250 µg/mL of (**a**) PME-H001, (**b**) PME-H002, (**c**) PME-H005, (**d**) PME-H007, and (**e**) PME-H008 fungus strain methanol extracts, as detailed in the text. Cell growth inhibition was evaluated via the MTT reduction assay and ODs measured at 570 nm, using 0.05 µg/mL vincristine sulphate (VC) as a positive control, as detailed in the text. VC caused 77.4%, 46.8%, 14.3%, 24%, and 14.7% cytotoxicity against L5178Y-R, HT-29, MCF-7, MA-104, and PBMCs, respectively. Data represent mean ± SEM of triplicates from three independent experiments. * *p* < 0.05; ** *p* < 0.01, as compared with untreated control. OD for untreated control was 1.39 ± 0.12.

Fungal methanol extracts were evaluated at concentrations ranging from 31 µg/mL to 250 µg/mL against the tumor cell lines L5178Y-R, HT-29, and MCF-7, and the normal cells MA-104 and PBMCs. PME-H001, PME-H002, PME-H005, PME-H007, and PME-H008 filamentous fungus strains showed significant (*p* < 0.05) tumor cell growth inhibition (Figure 1). Extracts were effective against L5178Y-R cells, whereas the least susceptible cell line was HT-29. PME-H008 extract caused the highest growth inhibition percentage against L5178Y-R and HT-29 cells with 96.6% (*p* < 0.01) and 42.5% (*p* < 0.05) respectively, whereas PME-H007 extract caused the highest growth inhibition percentage (*p* < 0.01) against MCF-7 cells with 55.8% at 250 µg/mL. Regarding IC_50_, PME-H005 extract showed the lowest values with 95.21 µg/mL against the MCF-7 cell line, followed by PME-H008 with 101 µg/mL against L5178Y-R cells. In addition, extracts showed an IC_50_ higher than 291 µg/mL against the HT-29 cell line, whereas the lowest percentage of growth inhibition was observed for PME-H001 and PME-H002 extracts with respective IC_50_ values of 437.7 µg/mL against MA-104 cells and 409.8 µg/mL against PBMCs (Figure 1). In addition, PME-H008 extract showed the highest SIs against L5178Y-R cells with values of 2.4 and 2.9, compared with MA-104 and PBMCs, respectively, whereas PME-H005 extract showed SIs of 2.77 and 1.5 against MCF-7 and L5178Y-R cells, respectively, compared with PBMCs (Table 2). IC_50_ values outside the highest concentration used (250 μg/mL) shown in Table 2 were determined using the Prisma software v.9. In this regard, when extract concentration that induces 50% growth inhibition was not reached or it may be found outside the highest concentration tested, this software facilitates its calculation. We did not evaluate higher than 250 μg/mL concentrations of the extracts due to their limited availability.

### 3.3. Molecular Identification of L. marginatus Endophytic Fungi with Cytotoxic Activity

We performed molecular identification of endophytic fungi that showed cytotoxic activity against the tumor cell lines L5178Y-R, HT-29, and MCF-7 (Figure 1). The amplified regions (ITS1/ITS4) were sequenced, manually reviewed, and analyzed using the Blast tool for fungal identification. PME-H001 and PME-H002 isolates were identified as *Penicillium citricum* with 99.6% and 99.2% identity, respectively (Table 3). PME-H005 and PME-H008 were only identified up to the genus level as *Aspergillus* sp. with 99% and *Cladosporium* sp. with 97.6%, whereas PME-H007 was identified as *Metarhizium anisopliae* with 98.9% identity (Table 3).

## 4. Discussion

Endophytic fungi represent an important source of biologically active compounds, including phenolic acids, alkaloids, quinones, steroids, saponins, tannins, and terpenoids with antidiabetic, anti-inflammatory, antiviral, immunosuppressive, anti-arthritis, antioxidant, antimicrobial, and anticancer effects [23]. However, less than 16% of fungal species have been cultured and studied and less than 5% of them represent important sources of bioactive metabolites [24]. The distribution of certain populations of endophytic fungi is restricted to a species or a family of plants, as well as to the genotype of the species. Thus, the presence of a specific population of fungi may determine the production of a variety of secondary metabolites [25]. Medicinal plants harbor endophytic fungi with the potential to produce pharmaceutically important products [26] due to the continuous metabolic interaction between fungus and plant, which may result in the production of similar compounds following similar metabolic pathways. For this reason, it is important to select plant species for the isolation of endophytic fungi in search of bioactive agents [27].

Therefore, this study reports the cytotoxic activity of endophytic fungi isolated from the medicinal plant *L. marginatus*, a species of cactus endemic to Mexico, which was previously reported to possess antitumor potential [16,17,18]. In the present study, we isolated *Penicillium*, *Aspergillus*, and *Cladosporium* genera, which are commonly obtained from plants that inhabit dry environments, such as cacti [19,28,29], whereas *Metarhizium* has been reported as a natural endophyte of *Glycine* [30], *Taxus* [31], *Brassica*, *Secale*, and *Avena* genera [32]. This study also showed *Metarhizium* as an endophyte of cacti.

Anticancer resistance is a serious problem in oncology, as in the case of breast cancer [33], colon cancer [34], and non-Hodgkin lymphoma [35]. Therefore, it is essential to search for and identify new compounds with cytotoxic activity, against which cancer cells are not resistant [36]. Various endophytic fungi have shown anticancer effects against hepatoma (HepG2), lung cancer (A-549), colorectal cancer (HCT-116, HT-29), breast cancer (MCF7), ovarian cancer (SKVO3), leukemia (HL-60), carcinoma (KB), cervical cancer (Hela), and lymphoma (L5178Y) [4].

The isolated strains of *P. citrinum*, PME-H001 and PME-H002, showed comparable cytotoxic activity, probably due to the production of similar compounds, however the metabolite profiles must be analyzed for confirmation. On the other hand, the anticancer activity of this species has been reported against different tumor cell lines, including A549, Hela, HepG2, L5178Y, MOLT-4, MCF-7, BT-474, and MDA-MB-231 and different compounds have been identified as being responsible for this activity, such as penicillocitrin A, 2-(2-acetyl-hydrazinyl) benzoic acid, 2-pyruvoylaminobenzamide, secalonic acid A, citriquinochroman, pyrrolidine alkaloids, pencitrin, and penicitrinone E [37,38,39,40,41]. In our study, *Cladosporium* sp. methanol extract caused moderate cytotoxicity against MCF-7 cells, whereas a report by Raj et al. [42] showed the activity of taxol obtained from *C. oxysporum* extracts against the T47D breast cancer cell line, with an IC_50_ value of 2.5 μM after 24 h of incubation.

Fungi of the genus *Aspergillus* are considered important sources of bioactive compounds with anticancer activity, among which are alkaloids, pyrones, polyketides, lactones, sterols, xanthones, anthraquinones, terpenes, peptides, depsipeptides, cyclic peptides, cytochalasins, enzymes, and proteins. They have been evaluated in different tumor cell lines such as MCF-7, HL-60, K-562, A549, MOLT-4, and HEP-G2 [43]. Furthermore, the potential of *M. anisopliae* to produce anticancer compounds such as taxol, with yields of 846.1 µg/L in liquid medium [31], and destruxin B, with IC_50_ values of 4.9 µM in A549 lung cancer cells, has been previously demonstrated [44].

Most anticancer drugs affect cancer and normal cells. Thus, researchers attempt to develop new drugs that are selective for cancer cells with minimal effects on other cells [5]. Determining the SI value results an essential tool for evaluating potential antitumor agents with limited toxicity to normal cells [45]. For evaluating any anticancer activity of a sample, its cytotoxicity against nonmalignant cells must be determined through the SI [45,46]. In our study, PME-H008 extract showed SI values of 2.4 and 2.9 against L5178Y-R cells, compared with MA-104 and PBMCs respectively, whereas SIs of PME-H005 extract against MCF-7 and L5178Y-R cells were 2.77 and 1.5 respectively, as compared with PBMCs (Table 2), which may represent prospective anticancer samples that warrant further investigation [45,47]. Endophytic fungi may play an important role in providing chemotherapeutic compounds with high specificity and minimal side effects.

## 5. Conclusions

The search for endophytic fungi from different habitats may provide an opportunity to discover new drugs and their application in human diseases. Evaluation of *L. marginatus* endophytic fungi methanol extracts have revealed their potential as producers of bioactive compounds with antitumor potential.

## Figures and Tables

**Table 1:** Morphological characterization and extract yields of *L. marginatus* endophytic fungi with cytotoxic activity.

Isolate Code	Radial Growth (mm)		Shape	Edge	Mycelium	Topside Color *	Underside Color *	Extract Yield
	3 d	7 d						
PME-H001	7.3	9.9	Circular	Filamentous	Flat	# 838B83	#F0E68C/EEF3E2	5.2%
PME-H002	8.5	17.5	Circular	Filamentous	Flat	#838B83	#F0E68C/EEF3E2	5.2%
PME-H005	8.1	17.9	Circular	Irregular	Flat	#838B83	#FFC125/FEF0C9	12%
PME-H007	6.2	22.6	Circular	Filamentous	Flat	#006400/FFFFFF	#CD9B10/EEDC82	8.9%
PME-H008	5.1	15.2	Circular	Entire	Flat	#2F4F4F/EBECE4	#FEFEF2	5.6%

* The colors of the colonies were defined using the color chart on the webpage http://www.webusable.com/coloursTable.htm (accessed on 9 February 2021).

**Table 2:** IC_50_^a^ and SI of tumor cell lines and MA-104 and PBMCs treated with fungus methanol extracts.

Isolate Code.	L5178Y-R		HT-29		MCF-7		MA-104		PBMCs	
	IC_50_^a^	SI *	IC_50_	SI	IC_50_	SI	IC_50_	SI	IC_50_	SI
PME-H001	269.4 ± 1.4	1.6/1 ^b^	348.1 ± 1.1	1.2/0.8	1387 ± 0.7	0.3/0.2	437.7 ± 0.8	1	295.4 ± 1.2	1
PME-H002	266.5 ± 1.4	1.1/1.5	402.5 ± 1	0.7/1	1244 ± 0.6	0.2/0.3	295.4 ± 1.4	1	409.8 ± 1.2	1
PME-H005	166.2 ± 1.8	0.7/1.5	291.6 ± 1.2	0.4/0.9	95.21 ± 1	1.2/2.77	123.5 ± 1.3	1	264 ± 1.5	1
PME-H007	132.9 ± 1.5	1.8/0.7	291.7 ± 1.3	0.8/0.7	114.7 ± 1.3	2.1/1.8	245.9 ± 1.9	1	215.8 ± 1.6	1
PME-H008	101 ± 1.5	2.4/2.9	301.1 ± 1.2	0.8/0.9	337.5 ± 1.3	0.7/0.8	250.2 ± 1.2	1	298.8 ± 1.4	1

^a^ Values are provided in µg/mL; ^b^ SI of MA-104/PBMC compared with fungus strain extracts; * SI = IC_50_ of MA-104 or PBMC/IC_50_ of tumor cell lines.

**Table 3:** Molecular identification based on ITS1/TS4 sequences of cytotoxic *L. marginatus* endophytic fungi.

Isolate Code	Closest Relatives in NCBI	Query Cover	E-Value	Percent Identity	Classification
PME-H001	*Penicillium citrinum* strain *IBB_40* (MH793859.1)	97%	0.0	99.6%	*Penicillium citrinum*
PME-H002	*Penicillium citrinum* strain MEBP0016 (MT597829.1)	100%	6 × 10^−131^	99.2%	*Penicillium citrinum*
PME-H005	*Aspergillus tabacinus* (MT635280.1)	100%	2 × 10^−47^	99%	*Aspergillus* sp.
	*Aspergillus versicolor* strain HM65 (MT609910.1)	100%	2 × 10^−47^	99%	
PME-H007	*Metarhizium anisopliae* isolate CENIEN041 (HQ722915.1)	98%	0.0	98.9%	*Metarhizium anisopliae*
PME-H008	*Cladosporium* sp. isolate *978-SAB SA4 2* (MT820353.1)	98%	0.0	97.6%	*Cladosporium* sp.

## Data Availability

The datasets generated and/or analyzed during the present study are available from the corresponding author on reasonable request.
